# Supplementary material for: Survey of Colistin Resistance in Commensal Bacteria from Penaeus vannamei Farms in China
Source: Foods. 2023 May 26;12(11):2143. doi: 10.3390/foods12112143 (PMC10252472; doi:10.3390/foods12112143)
Supplement: Supplementary file 1 [file foods-12-02143-s001.zip › foods-2383239-supplementary.pdf]

Supplementary Data

Survey of colistin resistance in commensal bacteria from *Penaeus vannamei* farms in China

Yilin Zhang [1], Xinrui Lv [1], Weiwei Cao [2], Huang Zhang [3], Lei Shi [1,4], Weibin Bai [1] and Lei Ye [1,*]

[1] Institute of Food Safety and Nutrition, Jinan University, Guangzhou 510632, China; stzhyl@163.com (Y.Z.); lxrui_1995@163.com (X.L.); shilei@jnu.edu.cn (L.S.); baiweibin@163.com (W.B.)

[2] School of Food Science and Biology, Guangdong Polytechnic of Science and Trade,
Guangzhou 510640, China; weiwei09201029@163.com

[3] Guangzhou Double Helix Gene Technology Co., Ltd., Guangzhou International Bio Island Co., Ltd.,
Guangzhou 510320, China; 18680288520@163.com

[4] Shandong Yuwang Ecological Food Industry Co., Ltd., Yucheng 251200, China

* Correspondence: yelei@jnu.edu.cn; Tel.: +86-20-85220217

Table S1. *Bacillus. licheniformis* MLST primer information [53]

Gene	Primer	Sequence(5'to3')	Annealing temperature (°C)	Amplicon size (bp)
adk	*adk*-F	GGT AAA GGG ACA CAG GCT GA	58	465
	adk-R	TCG AGT AAA GGC TGG GTT TG		
ccpA	*ccpA*-F	TAT GAT GTA GCA CGC GAA GC	58	561
	ccpA-R	TAT CCC CAA GCG CTC TTT TA		
recF	*recF*-F	ACG GTT CTG TTC CCA TTC AG	58	561
	recF-R	CAT CAC GGC CAT TGA CAT AG		
rpoB	*rpoB*-F	AGG TCA ACT AGT TCA GTA TGG ACG	58	495
	rpoB-R	AAG AAC CGT AAC CGG CAA CTT		
spo0A	*spo0A*-F	GAA GTG CTT GGT GTC GCA TA	58	558
	spo0A-R	TGT GTA GCC GAA AAG TGA CG		
sucC	*sucC*-F	GGG TCC CGA CGG CCA ACA AA	58	549
	sucC-R	GGC CGG TTC CCC TCC GTA GT		

Table S2. 16sRNA results of some drug-resistant bacteria

Species	Sample site				Total
	Guangdong	Fujian	Jjiangsu	Shandong	
Bacillus spp.	83	92	12	18	205
Enterobacter spp.	13	7	0	0	20
Staphylococcus spp.	4	15	0	1	20
Aeromonas spp.	9	7	4	0	20
Rothia amarae	0	0	2	0	2
Lactococcus garvieae	0	9	5	0	14
Vagococcus sp.	1	0	0	5	6
Arthrobacter sp.	0	0	0	1	1
Proteus	6	2	0	0	8
Other genera	8	36	0	1	45

Table S3. Antibiotic resistance spectra of all tested antibiotics for the 202 tested isolates

Name	Isolation source	Region	Antibiotic resistance spectra	MDR Index
shui14	pond water	China-Shandong	SXT,CTR,FEP,IPM,CIP,AM,CF,GM,MEM,TE,FOS,C	1
GD-23-T	soil	China-Guangdong	IPM,AM,GM,FEP,CTR,TE,SXT,CF,C	0.75
BS4	pond water	China-Fujian	CTR,AM,FEP,SXT,IPM,FOS,CF,C	0.67
BS12	aquafeed	China-Fujian	CTR,AM,FEP,SXT,IPM,FOS,CF,C	0.67
BS13	aquafeed	China-Fujian	CTR,AM,FEP,SXT,IPM,FOS,CF,C	0.67
BS40	aquafeed	China-Fujian	CTR,TE,FEP,SXT,IPM,FOS,CF,C	0.67
GD-HY-S-1-26	pond water	China-Guangdong	IPM,MEM,FOS,AM,GM,SXT,C	0.58
GD-GWCS28	pond water	China-Guangdong	IPM,AM,FEP,CTR,TE,SXT,CF,C	0.67
B-S-6	pond water	China-Guangdong	IPM,MEM,FOS,AM,CTR,TE,CF,C	0.67
BS38	aquafeed	China-Fujian	CTR,AM,FEP,SXT,FOS,CF,C	0.58
BS45	aquafeed	China-Fujian	CTR,AM,FEP,SXT,FOS,CF,C	0.58
tx2-8	shrimp	China-Fujian	CTR,AM,FEP,SXT,FOS,CF,C	0.58
tS41	pond water	China-Fujian	CTR,AM,FEP,SXT,FOS,CF,C	0.58
BS48	aquafeed	China-Fujian	CTR,TE,FEP,SXT,CF,FOS,C	0.58
TS47	pond water	China-Fujian	CTR,TE,FEP,AM,CF,FOS,C	0.58
tt30	soil	China-Fujian	CTR,TE,FEP,SXT,CF,IPM,C	0.58
tx1-18	shrimp	China-Fujian	CTR,AM,FEP,SXT,IPM,CF,C	0.58
tx2-51	shrimp	China-Fujian	CTR,AM,FEP,SXT,IPM,CF,C	0.58
tx2-44	shrimp	China-Fujian	CTR,TE,AM,FEP,SXT,CF,C	0.58
GD-15-T	soil	China-Guangdong	AM,FEP,CTR,TE,SXT,CF,C	0.58

GD-S-9	aquafeed	China-Guangdong	AM,FEP,CTR,TE,SXT,CF,C	0.58
GD-SL-20	aquafeed	China-Guangdong	AM,FEP,CTR,TE,SXT,CF,C	0.58
GD-37-T	soil	China-Guangdong	IPM,AM,FEP,CTR,SXT,C	0.5
GWCS23	pond water	China-Guangdong	FOS,CTR,AM,TE,FEP,C	0.5
BS39	aquafeed	China-Fujian	CTR,TE,AM,SXT,CF,C	0.5
TS58	pond water	China-Fujian	TE,AM,CIP,FOS,CF,C	0.5
tx1-54	shrimp	China-Fujian	CTR,FEP,SXT,IPM,CF,C	0.5
tx2-16	shrimp	China-Fujian	CTR,AM,SXT,IPM,CF,C	0.5
tx2-30	shrimp	China-Fujian	CTR,FEP,SXT,MEM,CF,C	0.5
tS42	pond water	China-Fujian	CTR,AM,FEP,SXT,CF,C	0.5
tt12	shrimp	China-Fujian	CTR,AM,FEP,SXT,CF,C	0.5
tx1-1	shrimp	China-Fujian	CTR,AM,FEP,SXT,CF,C	0.5
tx1-2	shrimp	China-Fujian	CTR,AM,FEP,SXT,CF,C	0.5
tx1-3	shrimp	China-Fujian	CTR,AM,FEP,SXT,CF,C	0.5
tx1-4	shrimp	China-Fujian	CTR,AM,FEP,SXT,CF,C	0.5
tx1-15	shrimp	China-Fujian	CTR,AM,FEP,SXT,CF,C	0.5
tx1-16	shrimp	China-Fujian	CTR,AM,FEP,SXT,CF,C	0.5
tx1-17	shrimp	China-Fujian	CTR,AM,FEP,SXT,CF,C	0.5
tx1-20	shrimp	China-Fujian	CTR,AM,FEP,SXT,CF,C	0.5
tx1-21	shrimp	China-Fujian	CTR,AM,FEP,SXT,CF,C	0.5
tx1-22	shrimp	China-Fujian	CTR,AM,FEP,SXT,CF,C	0.5
tx1-24	shrimp	China-Fujian	CTR,AM,FEP,SXT,CF,C	0.5

tx1-25	shrimp	China-Fujian	CTR,AM,FEP,SXT,CF,C	0.5
tx1-28	shrimp	China-Fujian	CTR,AM,FEP,SXT,CF,C	0.5
tx1-32	shrimp	China-Fujian	CTR,AM,FEP,SXT,CF,C	0.5
tx1-36	shrimp	China-Fujian	CTR,AM,FEP,SXT,CF,C	0.5
tx1-37	shrimp	China-Fujian	CTR,AM,FEP,SXT,CF,C	0.5
tx1-44	shrimp	China-Fujian	CTR,AM,FEP,SXT,CF,C	0.5
tx1-51	shrimp	China-Fujian	CTR,AM,FEP,SXT,CF,C	0.5
tx2-7	shrimp	China-Fujian	CTR,AM,FEP,SXT,CF,C	0.5
tx2-9	shrimp	China-Fujian	CTR,AM,FEP,SXT,CF,C	0.5
tx2-10	shrimp	China-Fujian	CTR,AM,FEP,SXT,CF,C	0.5
tx2-11	shrimp	China-Fujian	CTR,AM,FEP,SXT,CF,C	0.5
tx2-22	shrimp	China-Fujian	CTR,AM,FEP,SXT,CF,C	0.5
tx2-25	shrimp	China-Fujian	CTR,AM,FEP,SXT,CF,C	0.5
tx2-26	shrimp	China-Fujian	CTR,AM,FEP,SXT,CF,C	0.5
tx2-31	shrimp	China-Fujian	CTR,AM,FEP,SXT,CF,C	0.5
tx2-33	shrimp	China-Fujian	CTR,AM,FEP,SXT,CF,C	0.5
tx2-39	shrimp	China-Fujian	CTR,AM,FEP,SXT,CF,C	0.5
tx2-54	shrimp	China-Fujian	CTR,AM,FEP,SXT,CF,C	0.5
GD-3-T	soil	China-Guangdong	AM,FEP,CTR,TE,SXT,CF,C	0.58
JS-YHS52	aquafeed	China-Jiangsu	CTR,AM,FEP,CF,FOS,C	0.5
JS-YHS57	aquafeed	China-Jiangsu	CTR,AM,FEP,CF,FOS,C	0.5
TWS5	aquafeed	China-Jiangsu	CTR,TE,FEP,CF,FOS,C	0.5

JS-TWS15	aquafeed	China-Jiangsu	CTR,AM,FEP,CF,FOS,C	0.5
GD-20-T	soil	China-Guangdong	IPM,FEP,CTR,SXT,C	0.42
GD-T-1	soil	China-Guangdong	AM,FEP,CTR,SXT,CF,C	0.5
GD-SL-16	aquafeed	China-Guangdong	AM,FEP,CTR,SXT,CF,C	0.5
GD-41-T	soil	China-Guangdong	AM,SXT,FEP,CTR,C	0.42
GD-28-T	soil	China-Guangdong	AM,SXT,FEP,CTR,C	0.42
GWCS29	pond water	China-Guangdong	FOS,FEP,CTR,TE,C	0.42
GWCS33	pond water	China-Guangdong	FOS,FEP,CTR,TE,C	0.42
GWCS49	pond water	China-Guangdong	FOS,FEP,CTR,TE,C	0.42
GWCS16	pond water	China-Guangdong	FOS,FEP,CTR,TE,C	0.42
GWCS17	pond water	China-Guangdong	FOS,FEP,CTR,TE,C	0.42
GD-GWCT10	soil	China-Guangdong	FOS,FEP,CTR,TE,C	0.42
GD-50-T	soil	China-Guangdong	AM,FEP,CTR,SXT,C	0.42
TS56	pond water	China-Fujian	CTR,AM,FEP,FOS,C	0.42
TS40	pond water	China-Fujian	TE,AM,FOS,CF,C	0.42
tt36	soil	China-Fujian	TE,SXT,IPM,FOS,C	0.42
tt55	soil	China-Fujian	AM,FEP,SXT,FOS,C	0.42
tx1-49	shrimp	China-Fujian	AM,FEP,SXT,CF,C	0.42
tx2-36	shrimp	China-Fujian	AM,FEP,SXT,CF,C	0.42
tt7	soil	China-Fujian	SXT,IPM,MEM,FOS,C	0.42
tS18	pond water	China-Fujian	CTR,FEP,SXT,CF,C	0.42
tx1-23	shrimp	China-Fujian	CTR,FEP,SXT,CF,C	0.42

tx2-6	shrimp	China-Fujian	CTR,FEP,SXT,CF,C	0.42
tx2-15	shrimp	China-Fujian	CTR,FEP,SXT,CF,C	0.42
tx2-21	shrimp	China-Fujian	CTR,FEP,SXT,CF,C	0.42
SD-S20	pond water	China-Shandong	CTR,FEP,CF,FOS,C	0.42
GD-T-24	soil	China-Guangdong	AM,CTR,SXT,C	0.33
GD-T-33	soil	China-Guangdong	FEP,CTR,SXT,C	0.33
B-10-X-3	shrimp	China-Guangdong	FEP,CTR,SXT,C	0.33
GWCS14	pond water	China-Guangdong	FOS,AM.TE,C	0.33
GD-2-T	soil	China-Guangdong	FEP,CTR,SXT,C	0.33
GD-TS11	aquafeed	China-Guangdong	AM,FEP,CTR,C	0.33
GD-HY-33-T	soil	China-Guangdong	AM,GM,TE,C	0.33
GWCS47	pond water	China-Guangdong	FOS,FEP,TE,C	0.33
GWCS41	pond water	China-Guangdong	FEP,CTR,TE,C	0.33
GD-GWC10X-11	shrimp	China-Guangdong	AM,CTR,TE,C	0.33
B-5-X-11	shrimp	China-Guangdong	FOS,FEP,CTR,C	0.33
GWC9X-9	shrimp	China-Guangdong	FOS,CTR,TE,C	0.33
B-X-25-44	shrimp	China-Guangdong	IPM,MEM,AM,C	0.33
GD-HY-26-T	soil	China-Guangdong	FEP,CTR,SXT,C	0.33
GD-HY-54-T	soil	China-Guangdong	FEP,CTR,SXT,C	0.33
BS60	aquafeed	China-Fujian	FEP,SXT,CF,C	0.33
TS50	pond water	China-Fujian	FEP,CTR,CF,C	0.33

Ts20	pond water	China-Fujian	FEP,CTR,CF,C	0.33
TS53	pond water	China-Fujian	FOS,CTR,CF,C	0.33
TS31	pond water	China-Fujian	FOS,FEP,CF,C	0.33
TS37	pond water	China-Fujian	CTR,TE,AM,C	0.33
tt13	soil	China-Fujian	TE,AM,CF,C	0.33
tt3	soil	China-Fujian	TE,FOS,CF,C	0.33
tx1-45	shrimp	China-Fujian	CTR,AM,CF,C	0.33
JS-YHS25	aquafeed	China-Jiangsu	FEP,CF,FOS,C	0.33
0.5s29	aquafeed	China-Shandong	AM,TE,FOS,C	0.33
0.5s40	aquafeed	China-Shandong	AM,TE,FOS,C	0.33
0.5s45	aquafeed	China-Shandong	AM,TE,FOS,C	0.33
tu31	soil	China-Shandong	AM,TE,FOS,C	0.33
tu50	soil	China-Shandong	CTR,FEP,FOS,C	0.33
2x20	shrimp	China-Shandong	AM,TE,FOS,C	0.33
GD-HY-T-14	soil	China-Guangdong	FEP,CTR,C	0.25
GD-HY-T-19	soil	China-Guangdong	FEP,FOS,C	0.25
GWCS4	pond water	China-Guangdong	FOS,FEP,C	0.25
GD-SL-21	aquafeed	China-Guangdong	AM,FEP,C	0.25
GWCS26	pond water	China-Guangdong	MEM,FOS,C	0.25
GD-GWC5X-4	shrimp	China-Guangdong	CIP,AM,C	0.25
GWC10X-1	shrimp	China-Guangdong	FEP,CTR,C	0.25
GWCS12	pond water	China-Guangdong	FOS,TE,C	0.25

GWCS9	pond water	China-Guangdong	FOS,FEP,C	0.25
GD-GWCT11	soil	China-Guangdong	MEM,FEP,C	0.25
GWCT18	soil	China-Guangdong	FEP,TE,C	0.25
B-2-T-11	soil	China-Guangdong	FEP,MEM,C	0.25
GD-HY-53-T	soil	China-Guangdong	FEP,CTR,C	0.25
TS2	pond water	China-Fujian	CTR,CF,C	0.25
TS35	pond water	China-Fujian	CTR,CF,C	0.25
TS36	pond water	China-Fujian	FOS,CF,C	0.25
tS16	pond water	China-Fujian	CTR,TE,C	0.25
ts55	soil	China-Fujian	CTR,TE,C	0.25
tt17	soil	China-Fujian	CTR,FEP,C	0.25
tS48	pond water	China-Fujian	TE,FOS,C	0.25
tS10	pond water	China-Fujian	TE,FOS,C	0.25
ts25	aquafeed	China-Fujian	TE,CF,C	0.25
TS38	pond water	China-Fujian	AM,FOS,C	0.25
tt28	soil	China-Fujian	AM,FOS,C	0.25
HYS3-16	aquafeed	China-Guangdong	FEP,C	0.17
GD-HY-T-16	soil	China-Guangdong	FEP,C	0.17
GD-HY-T-40	soil	China-Guangdong	FEP,C	0.17
GD-SL-1-3	aquafeed	China-Guangdong	TE,C	0.17
GWC9X-8	shrimp	China-Guangdong	FOS,C	0.17
GWCS1	pond water	China-Guangdong	CTR,C	0.17

GWCS2	pond water	China-Guangdong	SXT,C	0.17
GWCT22	soil	China-Guangdong	FOS,C	0.17
GWCT17	soil	China-Guangdong	FOS,C	0.17
B-T-9	soil	China-Guangdong	TE,C	0.17
GD-HY-24-T	soil	China-Guangdong	FOS,C	0.17
GD-4-X-16	shrimp	China-Guangdong	FEP,C	0.17
GD-28-S	pond water	China-Guangdong	FEP,C	0.17
B-S-31	pond water	China-Guangdong	TE,C	0.17
GWCS39	pond water	China-Guangdong	CTR,C	0.17
GWCS43	pond water	China-Guangdong	FOS,C	0.17
GWC5X-3	shrimp	China-Guangdong	FOS,C	0.17
B-10-X-5	shrimp	China-Guangdong	TE,C	0.17
B-9-X-6	shrimp	China-Guangdong	TE,C	0.17
GWCS5	pond water	China-Guangdong	CTR,C	0.17
B-T-1	soil	China-Guangdong	TE,C	0.17
B-SL-F-33	aquafeed	China-Guangdong	TE,C	0.17
B-SL-F-44	aquafeed	China-Guangdong	TE,C	0.17
tS21	pond water	China-Fujian	FOS,C	0.17
tS19	pond water	China-Fujian	TE,C	0.17
tS23	pond water	China-Fujian	TE,C	0.17
tt9	soil	China-Fujian	TE,C	0.17
tS54	pond water	China-Fujian	TE,C	0.17

tS44	pond water	China-Fujian	AM,C	0.17
ts32	aquafeed	China-Fujian	GM,C	0.17
ts33	aquafeed	China-Fujian	CTR,C	0.17
TWS11	aquafeed	China-Jiangsu	FOS,C	0.17
TWS19	aquafeed	China-Jiangsu	AM,C	0.17
0.5s35	aquafeed	China-Shandong	TE,C	0.17
1s2	aquafeed	China-Shandong	FOS,C	0.17
1s15	aquafeed	China-Shandong	FOS,C	0.17
shui11	pond water	China-Shandong	FOS,C	0.17
SD-S12	pond water	China-Shandong	CF,C	0.17
shui30	pond water	China-Shandong	FOS,C	0.17
SD-S36	pond water	China-Shandong	CTR,C	0.17
2x37	shrimp	China-Shandong	TE,C	0.17
2x48	shrimp	China-Shandong	TE,C	0.17
2x56	shrimp	China-Shandong	TE,C	0.17
B-SL-F-8	aquafeed	China-Guangdong	C	0.08
B-S-19	pond water	China-Guangdong	C	0.08
B-S-8	pond water	China-Guangdong	C	0.08
GWC2X-5	shrimp	China-Guangdong	C	0.08
GWCS18	pond water	China-Guangdong	C	0.08
GD-SL-42	aquafeed	China-Guangdong	C	0.08
B-SL-F-25	aquafeed	China-Guangdong	C	0.08

BS2	aquafeed	China-Fujian	C	0.08
tt45	soil	China-Fujian	C	0.08
YHS2	aquafeed	China-Jiangsu	C	0.08
YHS3	aquafeed	China-Jiangsu	C	0.08
YHS4	aquafeed	China-Jiangsu	C	0.08
YHS19	aquafeed	China-Jiangsu	C	0.08
TWS9	aquafeed	China-Jiangsu	C	0.08

Table S4. *B. licheniformis* genomes were recovered from the GenBank database

Name	Isolation source	Region	ST
GCF_001587315.1	Mushroom soup	Netherlands	3
GCF_015845325.1	unknown	USA	1
GCF_000008425.1	unknown	unknown	1
GCF_000011645.1	unknown	unknown	1
GCF_018332815.1	Honey	Japan	1
GCF_018332835.1	Honey	Japan	1
GCF_016026835.1	unknown	unknown	3
GCF_016126875.1	chicken	China:Chengdu	3
GCF_020686805.1	leaf	Tunisia:Bulla-Regia	3
GCF_020686825.1	leaf	Tunisia:Bulla-Regia	3
GCF_020686845.1	leaf	Tunisia:Bulla-Regia	3
GCF_021045125.1	goat	South Africa	3
GCF_002272455.1	uncut heroin sample	Germany	3
GCF_002272365.1	uncut heroin sample	Germany	3
GCF_003474315.1	feces	China: Shenzhen	3
GCF_001642595.1	soil	Czech Republic:Nerartovice	3
GCF_003253815.1	adult feces	South Korea: Bundang	3
GCF_003606405.1	Maotai Daqu	China	3
GCF_003952865.1	Korean adult feces	South Korea: Seoul	3
GCF_006974025.1	unknown	unknown	3
GCF_002966955.1	High-salt fermented soybean	South Korea: Suwon	3
GCF_004103575.1	food	South Korea	3
GCF_007831015.1	human feces	Netherlands	3
GCF_001925055.1	unknown	Netherlands	3
GCF_002271465.1	uncut heroin sample	Germany	3
GCF_007830775.1	cheese	Netherlands	3
GCF_007830795.1	seawater	Netherlands	3

GCF_007831035.1	human feces	Netherlands	3
GCF_007831215.1	air sampler	Netherlands	3
GCF_007831435.1	Pig faeces	Germany	1
GCF_007831465.1	Pig faeces	Germany	3
GCF_007831495.1	soil	Netherlands	3
GCF_007831515.1	Pig faeces Germany, Farm, Slaughterpigs; not receiving antibiotics or Bacillus based products	Germany	3
GCF_007831575.1	soil	Denmark: Vigerslev	3
GCF_007831655.1	soil	Denmark: Vigerslev	1
GCF_007831695.1	soil	Denmark: Vigerslev	3
GCF_007831705.1	soil	Denmark: Vigerslev	3
GCF_007831875.1	Pig faeces Germany, Farm, Slaughterpigs; not receiving antibiotics or Bacillus based products	Germany	3
GCF_007832095.1	swine faeces	Germany	3
GCF_007832135.1	swine faeces	Germany	1
GCF_007832235.1	aborted bovine fetus, lung	Netherlands	3
GCF_007832295.1	potato pulp for cattle feeding	Netherlands	3
GCF_007832395.1	aborted bovine, placenta	Netherlands	3
GCF_008368515.1	Gergoush (Sudanese Bread snack) primary fermentation step	Sudan	3
GCF_009662395.1	soil in grass field, non-agriculture site	Denmark:Dyrehave	3
GCF_009965255.1	soil	Canada	3
GCF_007832345.1	aborted bovine fetus, lung	Denmark	3
GCF_010578005.1	commercial probiotic used for environment	China	1
GCF_014673515.1	Crude salt from Camalti Saltern	Turkey: Izmir	1
GCF_020686785.1	plant leaf	Tunisia: Bulla-Regia	3
GCF_023713085.1	Cleanroom	USA	3
GCF_023713165.1	Cleanroom	USA	3
GCF_023714845.1	Cleanroom	USA	3
GCF_001587355.1	dairy environment	Netherlands	3
GCF_000315975.1	Chinese Maotai-flavor liquor making process	China: Jiangsu	3
GCF_002272395.1	uncut heroin sample	Germany	3
GCF_000258125.2	soil from Yingcheng Salt Mine	China: Hubei Province	3

GCF_001507755.1	unknown	Netherlands	4
GCF_016629635.1	mining drainage	Peru: Lima	3
GCF_016902155.1	Gallusgallus' caecum	unknown	1
GCF_007832035.1	unknown	unknown	1
GCF_007994365.1	unknown	unknown	1
GCF_006094335.1	unknown	unknown	1
GCF_006494795.1	silt	China: Qinghai Province	3
GCF_900110715.1	unknown	unknown	3
GCF_007994305.1	swine faeces	Germany	3
GCF_007994355.1	unknown	Netherlands	1
GCF_008368415.1	Gergoush (Sudanese Bread snack) primary fermentation step	Sudan	3
GCF_008368425.1	Gergoush (Sudanese Bread snack) primary fermentation step	Sudan	3
GCF_902386215.1	Human gut	unknown	3
GCF_021045185.1	horse	South Africa	3
GCF_022630555.1	biocompost	Germany	3
GCF_022700915.1	unknown	China:Beijing	1

Table S5. Antibiotic resistance genes, pathogenicity score, virulence factors and mobile genetic elements of *Bacillus licheniformis*

Isolate	Resistance gene	Pathogenicity Score (no. of pathogenic family)	Virulence factors	Mobile genetic elements
FJ-ts47	ErmD, BcⅢ, qacJ,bcrA/B/C	0.79（59）	fliP,clpE,invA,cesA,ureB,mxiA,flhA	IS1182,IS3(IS150),IS1595(ISPna2),ISL3,IS5,ISNCY(ISDol1)
FJ-ts50	qacJ,ErmD,BcⅢ	0.808(266)	dhbF,clpP,lap,entB,lepA	IS1182, IS1595(ISPna2), IS3(IS150), IS1595(ISNha5)
FJ-ts53	qacJ,ErmD,BcⅢ	0.808(266)	dhbF,clpP,lap,entB,lepA	IS1182, IS1595(ISPna2),IS3(IS150),IS1595(ISNha5), ISAs1
FJ-ts58	qacJ,ErmD,BcⅢ,bcrA/B/C	0.79（59）	fliP,clpE,invA,cesA,ureB,mxiA,flhA	IS1182,IS1595(ISPna2),IS3(IS150), IS3(IS3),ISL3,IS5(IS5)
GD-GWCS28	ErmD, BcIII, qacJ,bcrA/B/C	0.81(62)	dhbF,clpP,lap,capB,pvdl,Cj1440c,icl,dhbE	IS1182,IS3(IS150),IS1595(ISPna2),IS1595(IS1595)ISL3,IS110
GD-GWC10X-1	ErmD, Bclll, qacJ	0.803(246)	fliP,invA,cpsA,flhB	IS1182,IS3(IS150),IS1595(ISPna2),IS3(IS3),IS630,IS256, ISAs1,ISNCY,IS110,IS4
GD-GWCT1	qacJ,ErmD,BcⅢ,bcrB/C	0.81(62)	dhbF,capB,pvdl,dhbE,pchD	IS1182,IS1595(ISPna2),IS3(IS150),ISL3,IS110,IS66,IS5(ISL2),IS1595(IS1595) ,Tn3
GD-TS11	qacJ,BcⅢ	0.807(230)	fliP,invA,cpsA,flhB,narH	IS1182, IS1595(ISPna2), IS3(IS150)
GD-GWC5X-4	qacJ,ErmD,BcⅢ	0.808(269)	fliP,clpE,invA,cpsA,flhB,lplAl	IS1182, IS1595(ISPna2) ,IS3(IS150),IS1595(ISNha5),ISAs1
GD-GWCT10	qacJ,ErmD,BcⅢ,bcrA/B/C	0.81（62）	dhbF,clpP,lap,capB,pvdl,Cj1440c,icl,dhbE	IS1182, IS1595(ISPna2),IS3(IS150), ISL3, IS1182, IS110,IS1595,Tn3
JS-TWS15	qacJ,ErmD,BcⅢ	0.808(227)	clpE,lplAl	IS1182,IS1595(ISPna2),IS3(IS150)
JS-YHS25	ErmD, Bclll, qacJ	0.808(266)	dhbF,clpP,lap,entB,lepA	IS1182,IS3(IS150),IS1595(ISPna2),IS1595(ISNha5) ,ISAs1
JS-YHS52	qacJ,ErmD,BcⅢ	0.808(265)	dhbF,clpP,lap,entB,lepA	IS1182, IS1595(ISPna2), IS3(IS150), IS1595(ISNha5),ISAs1
JS-YHS57	qacJ,ErmD,BcⅢ	0.808(267)	dhbF,clpP,lap,entB,lepA	IS1182, IS1595(ISPna2), IS3(IS150), IS1595(ISNha5),ISAs1
SD-S12	qacJ,ErmD,BcⅢ	0.808(266)	dhbF,clpP,lap,entB,lepA	IS1182, IS1595(ISPna2), IS3(IS150), IS1595(ISNha5),ISAs1
SD-S20	ErmD, Bclll, qacJ	0.808(266)	dhbF,clpP,lap,entB,lepA	IS1182,IS3(IS150),IS1595(ISPna2),IS1595(IS1595),ISL3,IS110
SD-S36	qacJ,ErmD,BcⅢ	0.808(266)	dhbF,clpP,lap,entB,lepA	IS1182, IS1595(ISPna2), IS3(IS150), IS1595(ISNha5)
